# Investigating salivary growth factor responses to tooth extraction in patients with type 2 diabetes: a prospective observational comparative study

**DOI:** 10.3389/froh.2026.1732241

**Published:** 2026-04-17

**Authors:** Mohammed Amjed Alsaegh, Yousuf Ibrahim Al Shehhi, Shishir Ram Shetty, Sam Thomas Kuriadom, Okba Mahmoud, Jayaraj Kodangattil Narayanan, Sudhir Rama Varma

**Affiliations:** 1 Department of Oral and Craniofacial Health Sciences, College of Dental Medicine, University of Sharjah. Sharjah, United Arab Emirates; 2 Research Institute for Medical and Health Sciences, University of Sharjah. Sharjah, United Arab Emirates; 3 Department of Clinical Sciences, College of Dentistry, Ajman University, Ajman, United Arab Emirates; 4 Center for Medical and Bio-allied Health Sciences Research, Ajman University, Ajman, United Arab Emirates; 5 Basic Medical and Dental Sciences Department, Ajman University, Ajman, United Arab Emirates

**Keywords:** diabetic, EGF, FGF, growth factors, PDGF, salivary cytokines, TGF-α, tooth extraction

## Abstract

**Objectives:**

This study aimed to evaluate salivary growth factor levels in controlled type 2 diabetic patients compared to healthy individuals, assessed at two time points: before tooth extraction and two days postoperatively.

**Materials and methods:**

The study included 27 participants: 20 with type 2 diabetes (74.07%) and 7 healthy controls (25.93%). Unstimulated whole saliva samples were collected before tooth extraction and two days afterward. Biomarker analysis was performed using a Luminex multiplex assay, targeting epidermal growth factor (EGF), transforming growth factor-alpha (TGF-α), fibroblast growth factor (FGF), platelet-derived growth factor-AB (PDGF-AB), platelet-derived growth factor-CC (PDGF-CC), and platelet-derived growth factor-DD (PDGF-DD).

**Results:**

EGF levels showed no significant differences between groups at baseline (*p* = 0.333) or post-extraction (*p* = 0.571); however, two days after extraction, EGF levels almost remained steady in diabetics but decreased in controls. TGF-α was not significantly different between diabetics and control groups at baseline and two days post-extraction (*p* = 0.088; *p* = 0.915, respectively), with levels increasing in diabetics and decreasing in controls two days after extraction. FGF levels showed no significant differences at baseline (*p* = 0.064) or post-extraction (*p* = 0.677), from baseline to second post-extraction day, the levels were increasing in diabetics and decreasing in controls. Between baseline and the second day post-extraction, the levels of PDGF-DD, PDGF-CC, and PDGF-AB decreased in both groups, except for PDGF-AB in the control group, where a slight increase was observed. No significant differences were found between the diabetic and control groups for PDGF-DD, PDGF-CC, or PDGF-AB at baseline (*p* = 0.810, *p* = 0.382, *p* = 0.881) or two days post-extraction (*p* = 0.860, *p* = 0.414, *p* = 0.740), respectively.

**Conclusions:**

Salivary growth factors are reduced and respond differently in type 2 diabetes, potentially contributing to impaired oral wound healing. Supplementing growth factors may improve clinical outcomes, warranting further investigation.

## Introduction

Tooth extraction is a common oral surgical procedure, and the healing of the extraction socket is the dentist's primary concern. An extraction socket heals in four stages: initial blood clot formation, inflammatory phase, woven bone formation, and remodeling (1). The healing process of extraction sockets is governed by numerous molecules and cytokines, involving both soft and hard tissues. The process includes osteocyte proliferation and differentiation, as well as the synthesis and mineralization of the extracellular matrix that results in bone formation (2).

Diabetes is a metabolic disorder characterized by impaired insulin secretion and function, which results in hyperglycemia and microvascular complications (3). Although some studies have not found definitive evidence linking diabetes to consistently delayed healing (4), patients with poorly controlled or untreated diabetes mellitus frequently experience delayed healing of tooth extraction sockets (5, 6). Prolonged wound healing in diabetic patients is typically linked to abnormal cellular function and disrupted regulation of growth factors and cytokines essential for coordinating the normal healing process (2).

Epidermal growth factor (EGF) promotes wound healing by suppressing inflammatory mediators and boosting the expression of genes linked to cell proliferation and angiogenesis. It also influences both mesenchymal and epithelial cells, aiding in the formation of new blood vessels and minimizing inflammation (7). Transforming growth factor-alpha (TGF-α) is a member of the EGF family and functions to stimulate the clustering and activation of inflammatory cells, as well as promote the release of inflammatory mediators (8). TGF-α, together with EGF, plays a role in oral wound healing. In a hamster model, eosinophils in oral wounds produced TGF-α in the absence of salivary EGF, indicating that salivary TGF-α and EGF have complementary functions in the healing process (9). Fibroblast growth factor (FGF) directs oral tissue repair by stimulating fibroblast proliferation, angiogenesis, re-epithelialization, extracellular matrix remodeling, and cell migration (10).

Platelet-derived growth factors (PDGFs) are inactive in their monomeric forms, consisting of four distinct polypeptide chains: PDGF-A, PDGF-B, PDGF-C, and PDGF-D. Their activation occurs through dimerization, resulting in homodimeric isoforms (PDGF-AA, PDGF-BB, PDGF-CC, and PDGF-DD) or a heterodimeric isoform (PDGF-AB) (11). PDGFs play a vital role in facilitating fibroblast migration and proliferation, processes essential for tissue repair and regeneration. Additionally, it stimulates angiogenesis, providing the necessary nutrients and oxygen to support tissue healing (12).

Saliva offers a practical, non-invasive medium that may reflect local and systemic alterations relevant to oral wound healing, including diabetes-associated changes (13). However, data describing peri-extraction dynamics of salivary growth factors in controlled type 2 diabetic patients, particularly in the early postoperative period, remain limited.

Dysregulation and altered levels of growth factors and cytokines in diabetic patients may contribute to impaired wound healing and are closely associated with the progression of diabetes and its complications (2, 14). This study aimed to evaluate the levels of salivary growth factors in controlled type 2 diabetic patients compared to healthy individuals, assessed at two distinct time points: prior to tooth extraction and two days postoperatively.

## Materials and methods

**Figure 1:**
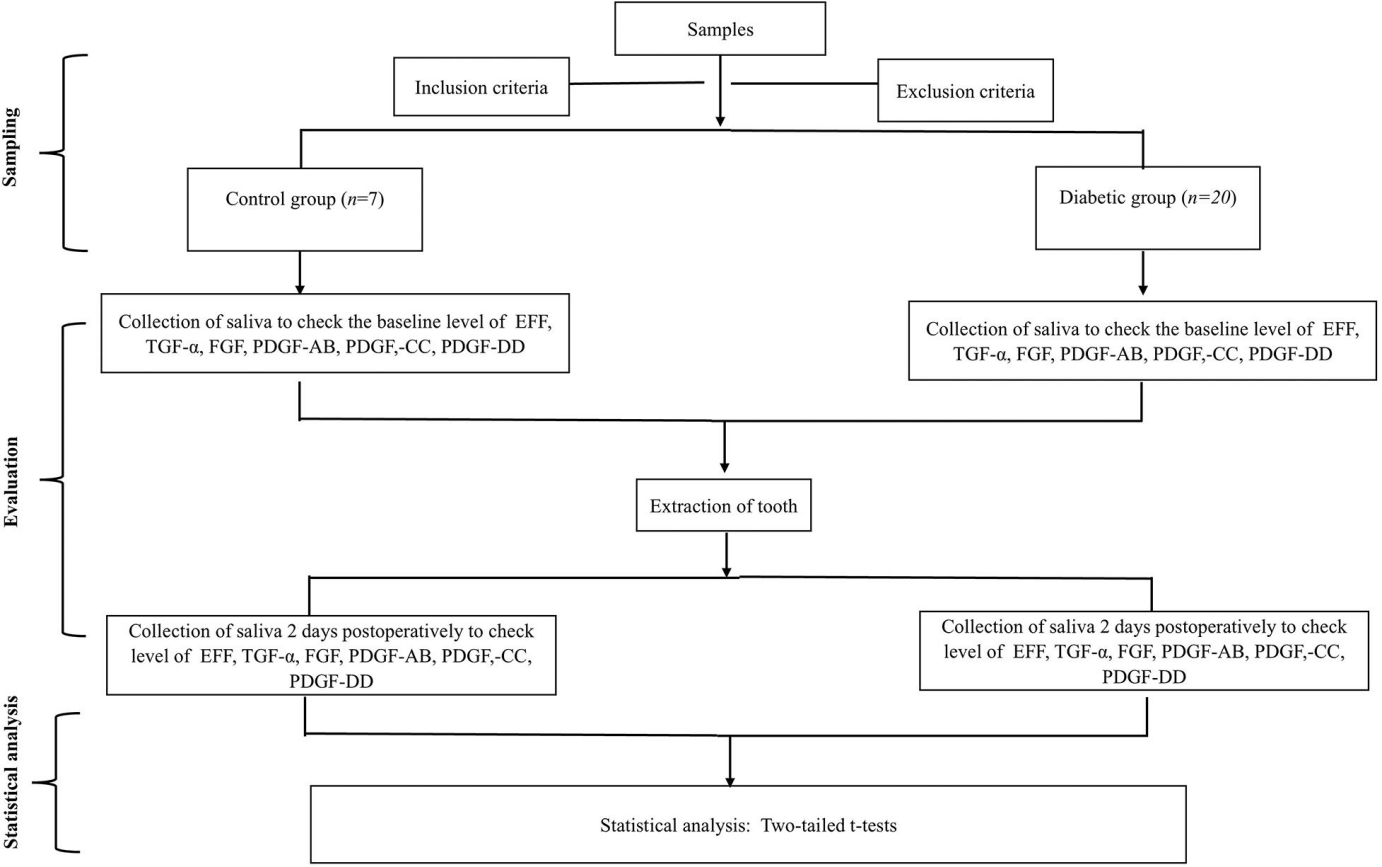
A diagrammatic representation of the chronology of the methodology.

The study flowchart is presented in Figure 1.

### Study design and participants

This prospective observational comparative study included 27 patients recruited from the University Dental Hospital of Sharjah from October 1, 2021, to September 21, 2022. Among them 20 participants (74.07%) had type 2 diabetes mellitus, and seven (25.93%) were healthy controls. The study included individuals aged over 18 who were scheduled for tooth extraction based on guidelines of oral surgery clinic. Participants had been diagnosed with type 2 diabetes mellitus for at least one year and demonstrated recent HbA1c levels below 7.5% and random glucose levels under 180 mg/dL.

All diabetic patients were controlled and free of diabetes-related systemic complications. Control participants reported no systemic diseases. Both groups were selected based on the indication for tooth extraction for non-restorable necrotic teeth without active periodontal or apical lesions. Exclusion criteria included smoking, any tobacco use, immunological conditions, immunosuppressive treatments, ongoing antibiotic or anti-inflammatory therapy, acute infections, pregnancy or breastfeeding, the need for prophylactic antibiotics for dental procedures, and any systemic conditions impacting the healing response.

Sample size was estimated using GPower (v3.1.9.7; Heinrich Heine University Düsseldorf, Düsseldorf, Germany). The *a priori* power analysis was based on the primary outcome, defined as the within-participant change in salivary EGF from baseline (pre-extraction) to 2 days post-extraction. Accordingly, a two-tailed paired-samples *t*-test was specified in GPower (*t*-tests; difference between two dependent means, matched pairs), with *α* = 0.05% and 80% power. Based on the planning parameters reported by Gutiérrez-Corrales et al. (15) and accounting for the paired design (Cohen's dz, calculated using the SD of the within-subject differences), the anticipated effect size was set at dz = 0.65, which indicated a minimum required sample of 21 participants. To accommodate potential attrition and sample loss, 27 participants were recruited. All participants provided informed consent, and the study adhered to the principles outlined in the Declaration of Helsinki. Ethical approval was obtained from the Human Research and Ethics Committee at the University of Sharjah (REC-21-06-01-03-S).

### Saliva sample collection

Two 5-mL samples of unstimulated whole saliva were collected from each patient: one prior to tooth extraction and another two days post-extraction. The 2-day post-extraction time point was chosen to capture the early postoperative phase, when inflammatory and early reparative responses change rapidly. This timing is also consistent with our previous post-extraction salivary biomarker analysis (13). Participants refrained from eating or drinking for one hour before sampling. They rinsed their mouths gently with water for two minutes before spitting into a collection tube for five minutes. Samples were stored on ice and subsequently frozen at −80 °C for analysis.

### Measurement of salivary biomarkers

Levels of salivary biomarkers (EGF, TGF-α, FGF, PDGF-AB, PDGF-CC, and PDGF-DD) were measured using a Luminex multiplex assay. Samples were brought to room temperature, centrifuged at 4,000 rcf for 15 min at 4 °C, and the supernatant was mixed with a calibrant diluent. The concentrations of cytokines were assessed with a Human Magnetic Pre-Mixed Analyte Luminex Assay (R&D Systems, UK). Standards were prepared according to the manufacturer's protocol with a 3-fold serial dilution, resulting in seven standards. Following preparation, 50 µL of standards and samples were added to a 96-well plate along with 50 µL of premixed microparticle cocktail, then incubated at room temperature on an orbital shaker. After washing with a 25-fold diluted buffer, 50 µL of a biotin-antibody cocktail was added and incubated. Streptavidin-PE was added subsequently, followed by a final washing and fluorescence detection on the Bio-Plex-200 system (Bio-Rad Laboratories, USA). The data were analyzed using Bio-Plex Manager software, and concentrations were calculated using a 5-parameter logistic curve.

### Statistical analysis

Data were collected, organized, and statistically analyzed using SPSS version 28 (IBM Corporation, Armonk, NY, USA). Quantitative variables are presented as means, standard deviations, coefficients of variation, ranges, and standard errors. A two-tailed independent-samples *t*-test was used for between-group comparisons, and a two-tailed paired-samples (dependent) *t*-test was used for within-group comparisons (baseline versus 2 days post-extraction). Normality was assessed using Shapiro–Wilk tests. Between-group comparisons were performed using independent-samples *t*-tests; homogeneity of variance was evaluated with Levene's test, and Welch's *t*-test (equal variances not assumed) was applied when Levene's test was significant (*p* < 0.05). Statistical significance was set at *p* < 0.05 (page 4).

## Results

This study examined the acute response of salivary growth factors at baseline and following simple tooth extraction in 27 individuals. Demographic details of participants are presented in Table 1.

**Table 1:** Demographic data of the study participants.

Characteristics	Total samples	Diabetes mellitus group	Control group
Number	27	20	7
Gender	Male = 21	Male = 16	Male = 5
	Female = 6	Female = 4	Female = 2
Mean age	51.67 ± 13.26	57.95 ± 7.48	33.71 ± 8.77
Hb A1C	6.381 ± 1.01	6.945 ± .22	4.771 ± .43
Random Blood glucose level	132.41 ± 17.53	138.40 ± 14.07	115.29 ± 15.61

### Salivary biomarker concentrations

#### EGF

**Figure 2:**
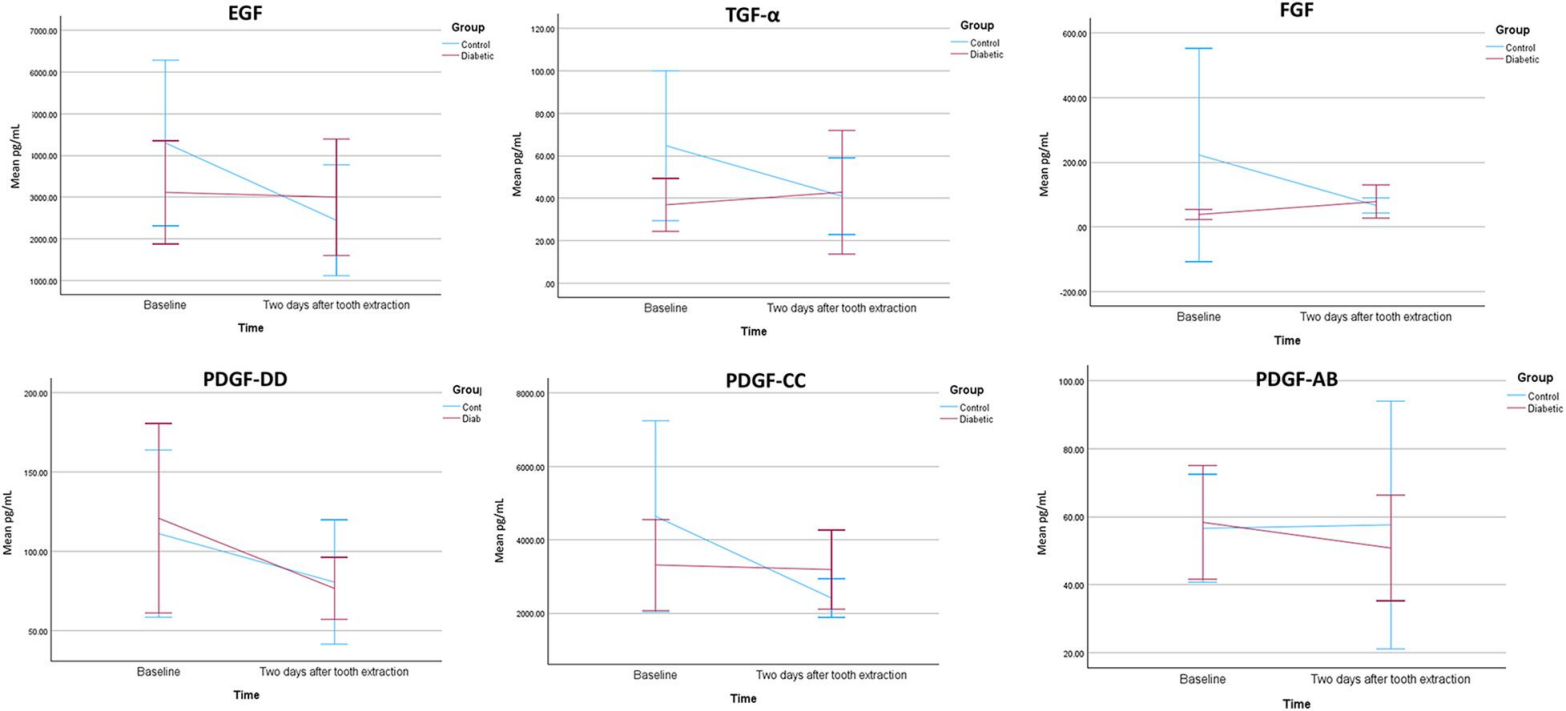
Line graphs showing salivary concentrations (pg/mL) of epidermal growth factor (EGF), transforming growth factor alpha (TGF-α), fibroblast growth factor (FGF), platelet-derived growth factor-AB (PDGF-AB), platelet-derived growth factor-CC (PDGF-CC), and platelet-derived growth factor-DD (PDGF-DD) measured at baseline (pre-extraction) and 48 h (2 days) after tooth extraction in individuals with type 2 diabetes and nondiabetic controls. Data points represent group means at each time point, connected by lines to illustrate the within-group change over time. Vertical error bars indicate variability around the mean and are plotted as plus or minus 2 standard errors (±2 SE).

In the diabetes mellitus group, the mean EGF concentrations were 3,113.86 pg/mL at baseline and 3,000.12 pg/mL two days post-extraction. For the non-diabetic group, baseline and post-extraction concentrations were 4,300.74 pg/mL and 2,443.26 pg/mL, respectively. No significant differences were observed between groups at either baseline or post-extraction (*p* = 0.333; *p* = 0.571, respectively) (Table 2). EGF levels remained relatively stable in the diabetes mellitus group after extraction, while the non-diabetic group exhibited a decrease in EGF two days post-extraction (Figure 2). There was no statistically significant difference in salivary EGF levels between the baseline measurement and two days postoperatively in both the diabetic and control groups (Table 3).

**Table 2:** Comparison of salivary EGF, TGF, FGF, PDGF-DD, PDGF-CC, and PDGF-AB levels between type-2 diabetic patients and control individuals at baseline and 48 h after tooth extraction.

No.	Marker	Group	Number	Baseline levels			Levels two days post-extraction		
				Mean ± SDpg/mL	t value	*P* value	Mean ± SDpg/mL	t value	*P* value
1	EGF	Diabetes mellitus	20	3,113.86 ± 2,768.49	1.01	.33	3,000.12 ± 3,128.03	−.58	.57
		Control	7	4,300.74 ± 2,632.22			2,443.26 ± 1,760.50		
2	TGF-α	Diabetes mellitus	20	36.89 ± 27.81	1.49	.09	42.82 ± 65.23	−.11	.96
		Control	7	64.75 ± 46.64			40.97 ± 23.90		
3	FGF	Diabetes mellitus Control	20	38.31 ± 36.05	1.94	.06	78.08 ± 114.808	−.42	.68
			7	222.51 ± 437.06			66.15 ± 31.61		
4	PDGF-DD	Diabetes mellitus	20	120.98 ± 133.72	-.24	.81	76.81 ± 43.71	.18	.86
		Control	7	111.29 ± 69.61			80.78 ± 51.85		
5	PDGF-CC	Diabetes mellitus	20	3,313.37 ± 2,773.15	.92	.38	3,189.48 ± 2,413.99	−.83	.41
		Control	7	4,639.88 ± 3,449.01			2,412.22 ± 695.17		
6	PDGF-AB	Diabetes mellitus	20	58.34 ± 37.41	-.15	.88	50.79 ± 34.83	.34	.74
		Control	7	56.59 ± 21.06			57.60 ± 48.30		

**Table 3:** Changes in salivary growth factor levels from baseline to two days post-extraction in control and diabetic patients.

	Control group				Diabetic group			
	Baseline levels	Levels two days post-extraction	t value	*P* value	Baseline levels	Levels two days post-extraction	t value	*P* value
EGF	4,300.74 ± 2,632.22	2,443.26 ± 1,760.50	2.12	.08	3,113.86 ± 2,768.49	3,000.12 ± 3,128.03	.17	.87
TGF-α	64.75 ± 46.64	40.97 ± 23.90	1.17	.29	36.89 ± 27.81	42.82 ± 65.23	−.36	.72
FGF	222.51 ± 437.06	66.15 ± 31.61	.93	.39	38.31 ± 36.05	78.08 ± 114.81	−1.39	.18
PDGF-DD	111.29 ± 69.61	80.78 ± 51.85	.81	.45	120.98 ± 133.72	76.81 ± 43.71	1.47	.16
PDGF-CC	4,639.88 ± 3,449.01	2,412.22 ± 695.17	1.65	.150	3,313.37 ± 2,773.15	3,189.48 ± 2,413.99	.18	.86
PDGF-AB	56.59 ± 21.06	57.60 ± 48.30	-.04	.97	58.34 ± 37.41	50.79 ± 34.83	.64	.53

#### TGF-α

Mean TGF-α concentrations in the diabetes mellitus group were 36.89 pg/mL before extraction and 42.82 pg/mL two days postoperatively. In the non-diabetic group, these levels were 64.75 pg/mL at baseline and 40.97 pg/mL post-extraction. No significant differences were observed between groups at either baseline or post-extraction (*p* = 0.088; *p* = 0.915, respectively) (Table 2). TGF-α levels increased in the diabetes mellitus group following extraction, while the non-diabetic group showed a decrease in its level (Figure 2). There was no statistically significant difference in salivary TGF-α levels between the baseline measurement and two days postoperatively in both the diabetic and control groups (Table 3).

#### FGF

Mean FGF levels in the diabetes mellitus group were 38.31 pg/mL at baseline and 78.08 pg/mL post-extraction. In the non-diabetic group, the levels were 222.51 pg/mL before extraction and decreased to 66.15 pg/mL two days postoperatively. No significant differences were found between groups at baseline or post-extraction (*p* = 0.064; *p* = 0.677, respectively) (Table 2). In the diabetes mellitus group, FGF levels increased post-extraction, whereas they decreased in the non-diabetic group (Figure 2). There was no statistically significant difference in salivary FGF levels between the baseline measurement and two days postoperatively in both the diabetic and control groups (Table 3).

#### PDGF-DD

The mean concentrations of PDGF-DD in the diabetes mellitus group were 120.98 pg/mL at baseline and 76.81 pg/mL two days post-extraction. In the non-diabetic group, levels were 111.29 pg/mL and 80.78 pg/mL at baseline and post-extraction, respectively. Differences between groups at baseline and post-extraction were not statistically significant (*p* = 0.810; *p* = 0.860, respectively) (Table 2). PDGF-DD levels decreased from baseline to post-extraction in both groups (Figure 2). There was no statistically significant difference in salivary PDGF-DD levels between the baseline measurement and two days postoperatively in both the diabetic and control groups (Table 3).

#### PDGF-CC

For the diabetes mellitus group, the mean PDGF-CC levels were 3,313.37 pg/mL at baseline and 3,189.48 pg/mL two days post-extraction. In the non-diabetic group, levels were 4,639.88 pg/mL at baseline and 2,412.22 pg/mL post-extraction. There were no significant differences between the groups at baseline or post-extraction (*p* = 0.382; *p* = 0.414, respectively) (Table 2). PDGF-CC levels remained stable in the diabetes mellitus group after extraction, while a decrease was observed in the non-diabetic group (Figure 2). There was no statistically significant difference in salivary PDGF-CC levels between the baseline measurement and two days postoperatively in both the diabetic and control groups (Table 3).

#### PDGF-AB

The diabetes mellitus group had mean PDGF-AB concentrations of 58.34 pg/mL at baseline and 50.79 pg/mL two days post-extraction. In the non-diabetic group, concentrations were 56.59 pg/mL at baseline and 57.60 pg/mL two days postoperatively. There were no significant differences between the groups at baseline or post-extraction (*p* = 0.881; *p* = 0.740, respectively) (Table 2). PDGF-AB levels exhibited a slight increase in the control group and a decrease in the diabetic group from baseline to two days post-extraction (Figure 2). There was no statistically significant difference in salivary PDGF-AB levels between the baseline measurement and two days postoperatively in both the diabetic and control groups (Table 3).

## Discussion

This study investigated salivary growth factor responses at baseline level and after tooth extraction in diabetic compared to non-diabetic patients. The findings revealed that baseline levels of EGF, TGF-α, FGF, and PDGF-CC were higher in non-diabetic individuals but tended to decrease two days post-extraction. In contrast, these levels remained generally stable in diabetic patients. PDGF-DD exhibited a similar response pattern in both groups. However, PDGF-AB levels slightly increased in the control group but decreased in the diabetic group two days post-extraction.

### Impact of diabetes on extraction socket healing

The healing of tooth extraction sockets is slower in diabetics than in those without diabetes (5, 6). Understanding the mechanisms underlying delayed wound healing after tooth extraction and exploring potential treatments is crucial, particularly due to the unique challenges posed by diabetes (2). Additionally, complications such as swelling and infection were more prevalent in the diabetic group (6). Diabetes has been linked to various macrovascular and microvascular complications, with notable alterations in microvascular circulation. These changes impair the inflammatory response, reducing leukocyte migration, tissue perfusion, and hyperemia. As a result, nutrient delivery and the removal of metabolic by-products are compromised (16).

### Lower baseline biomarker levels and altered response dynamics were observed in the diabetes group vs. controls

Diabetic patients often exhibit altered levels of various growth factors and cytokines, which are associated with the progression of diabetes and its complications (14). Growth factors found in saliva, such as EGF, are believed to contribute to oral health by supporting wound healing and preserving mucosal integrity (17). A reduction or alteration in these factors could subsequently affect the normal wound healing of the oral cavity. It was hypothesized that reduced cell proliferation at the injury site in diabetes, likely resulting from lower levels of growth factors, may adversely impact fracture and wound healing (18).

To the best of our knowledge, this is the first study to investigate salivary growth factors and their acute response to surgical trauma, such as tooth extraction, in diabetic patients.

It is widely acknowledged that biomarker levels in diseased conditions often vary from those seen in the healthy general population. The observation from the present study, which shows that the baseline levels of almost all studied biomarkers were numerically lower in individuals with diabetes than in controls, underscores the metabolic and physiological disturbances caused by the disease. These lower baseline levels suggest a disruption in homeostasis, likely due to chronic hyperglycemia and its associated complications. Diabetes is associated with increased levels of systemic inflammation and oxidative stress, both of which can downregulate the production of growth factors. Chronic inflammation can alter the normal regulatory pathways involved in growth factor synthesis, reducing their availability in bodily fluids like saliva (16). Understanding the deficiency in salivary growth factors in diabetic patients opens potential therapeutic avenues. For example, topical or systemic treatments to replenish these growth factors could be explored as a means of improving oral and systemic health outcomes in diabetic individuals.

The findings of the present study suggest that, following tooth extraction, the salivary levels of nearly all examined growth factors remain stable in diabetic patients, whereas they decrease in non-diabetic individuals. One could hypothesize that this relative stability reflects a compensatory mechanism that preserves growth factor availability in diabetic patients during the healing process. Alternatively, it could suggest reduced utilization of these essential growth factors in diabetic individuals. This deviation from the normal salivary growth factor response in diabetic patients highlights the significant impact of diabetes on growth factor regulation and wound healing. Gaining insight into these differences could guide the development of more targeted strategies for managing oral health in diabetic patients.

In healthy individuals, the wound healing process moves efficiently from the inflammatory phase to the proliferative phase, where tissue repair is active, and finally to the remodeling phase. During the proliferative phase, typically starting after the first 48 h, the requirement for high concentrations of growth factors diminishes as new tissue begins to form, and healing advances towards maturation. This transition may lead to a natural decrease in the levels of growth factors in saliva, which may no longer be needed in high concentrations during normal wound healing of healthy individuals. Correspondingly, research on wound healing shows that once the initial repair processes are underway, the body downregulates the production of growth factors like EGF and FGF to prevent excessive tissue formation and ensure proper wound closure (19). In fact, normal physiological responses to minor wounds, such as dental extractions, tend to involve rapid epithelial and connective tissue regeneration, decreasing the need for prolonged growth factor production. Additionally, it can be hypothesized that healthy individuals exhibit a more pronounced early utilization of growth factors within the extraction socket, contributing to lower salivary levels at 48 h, whereas diabetes may be associated with a blunted or altered utilization pattern over the same interval. This observation aligns with a prior study indicating that diabetes impairs the normal inflammatory response (13).

Growth factors like FGF, TGF-β, and EGF are critical for tissue repair by promoting angiogenesis, cell proliferation, and modulating inflammation (20). In diabetic patients, however, these growth factors may be less effectively utilized due to the impaired microvascular circulation, chronic inflammation, and oxidative stress associated with diabetes, which delays the wound healing process (20).

The distinct response of PDGF-AB and PDGF-DD, compared to the other growth factors analyzed in this study, may be linked to their specific timing and role during various phases of the healing process. Studies suggest that PDGFs exert prolonged effects relative to other growth factors, making them especially effective in managing chronic wound conditions commonly seen in diabetes (21). While growth factors that induce keratinocyte migration like EGF and FGF are produced in the early phases of healing (22, 23).

### The salivary EGF response after tooth extraction in diabetic vs. control groups

The current study revealed that while there were no significant differences in EGF levels between diabetic and healthy individuals at baseline or two days after tooth extraction, baseline EGF levels were numerically lower in the diabetic group. Two days after extraction, EGF levels remained stable in the diabetic group but showed a decrease in the control group. In agreement with our results, a previous study has reported lower salivary EGF levels in diabetic patients, suggesting that this reduction may contribute to the development of oral and systemic complications associated with diabetes (17). Similarly, Serrero et al. observed a decrease in salivary EGF levels in obese animal models (24). In contrast, other studies reported an increase in salivary EGF concentrations in diabetic patients (25, 26) alongside a decrease in their plasma EGF levels (25).

EGF plays a crucial role in wound healing, and a reduction in salivary EGF levels may impair oral wound healing (20), such as in the case of tooth extraction socket wounds. This is supported by a study showing that supplementing EGF in drinking water enhances the healing of diabetic oral wounds, while having minimal impact on the healing of non-diabetic oral wounds (22). Additionally, in a tongue mucosal wound healing model, the removal of submandibular salivary glands, which resulted in a confirmed reduction in salivary EGF content, was shown to hinder the healing process (22).

### The salivary TGF response after tooth extraction in diabetic vs. control groups

TGF-α, in conjunction with EGF, plays a critical role in oral wound healing. In a hamster model, eosinophils were shown to produce TGF-α in the absence of salivary EGF, highlighting the complementary roles of TGF-α and EGF in the healing process (9). TGF-α and TGF-β1, while both belonging to the TGF family, exhibit distinct and sometimes opposing functions. TGF-α primarily serves as a mitogen, promoting cell proliferation and survival, whereas TGF-β1 is associated with growth inhibition, cellular differentiation, and apoptosis. However, their roles can intersect in certain contexts, particularly in cellular survival and signaling pathways (27).

In the present study, baseline TGF-α levels were numerically lower in the diabetic group compared to the non-diabetic group. Two days post-extraction, TGF-α levels in the diabetic group showed increase, while the control group experienced a decrease in TGF-α levels over the same period. Previous research found that applying TGF-α to wounds in diabetic animals significantly improved wound closure compared to vehicle treatment, underscoring the therapeutic potential of these factors in managing diabetic wound healing (28).

### The salivary FGF response after tooth extraction in diabetic vs. control groups

The FGF family consists of structurally similar, multifunctional signaling molecules that are essential in regulating numerous developmental, physiological, and pathological processes in humans (29). Notably, FGFs play a significant role in angiogenesis, wound healing, embryonic development, and metabolism through both the paracrine and endocrine pathways (30). They also promote the proliferation of fibroblasts, which contribute to the formation of granulation tissue. This tissue fills the wound space and cavity during the initial stages of the wound healing process (10, 31).

The roles of most FGFs in diabetes are still not well explored (32). An elevated concentration of FGF-21 may reduce the risk of developing type 2 diabetes, while type 2 diabetes itself did not have a significant effect on FGF-21 levels (33).

The present study demonstrated that healthy individuals had higher baseline salivary FGF levels compared to those with type 2 diabetes. Additionally, FGF levels increased in the diabetic group two days after extraction, while the control group experienced a sharp decline over the same period. In agreement with our findings, previous research has suggested that low serum FGF-19 levels are positively associated with the development of type 1 diabetes mellitus and inversely correlated with fasting blood glucose levels (34). Moreover, immunohistochemical analysis in rats demonstrated that two weeks after the induction of bone distraction osteogenesis, the diabetic group exhibited significantly reduced FGF-2 expression in the osteotylus compared to the control group (35). Furthermore, research indicates that FGF13 levels are lower in individuals with impaired glucose tolerance and type 2 diabetes mellitus compared to healthy controls, suggesting its potential as a biomarker for pre-diabetes and type 2 diabetes (36). Similarly, FGF19 levels are reported to be lower in type 1 diabetic children compared to healthy controls, indicating a possible role in diabetes-related complications (37). Conversely, other studies reported that elevated circulating FGF-21 levels are positively associated with diabetes, with patients with type 2 diabetes showing significantly higher plasma FGF-21 levels (38, 39).

Interestingly, FGF-based drugs have been shown to effectively regulate several diabetic nephropathy processes, including inflammation, glucose and lipid metabolism, oxidative stress, and kidney injury. However, their use is limited by challenges such as insufficient pharmacokinetic data, a very short half-life, and the need for frequent administration (29).

### The salivary PDGF isoform response after tooth extraction in diabetic vs. control groups

PDGF promotes the synthesis of new extracellular matrix components and acts as a mitogen for fibroblasts. Additionally, it drives the differentiation of fibroblasts into myofibroblasts, aiding in collagen matrix contraction and wound healing during the proliferation phase (31). PDGF was the first recombinant growth factor to receive approval for topical use to expedite wound closure (31).

In the current study, we observed varying responses among different types of salivary PDGF in diabetic patients compared to healthy individuals. PDGF-AB and PDGF-DD exhibited nearly similar baseline levels and responded differently to tooth extraction compared to the other salivary growth factors analyzed between the control and diabetic groups. Nevertheless, PDGF-CC exhibited a response pattern resembling that of EGF, TGF-α, and FGF, marked by lower baseline levels and a more stable response over two postoperative days in diabetic patients compared to the control group. This underscores the complexity of growth factor regulation in diabetes and highlights the potential of saliva as a diagnostic tool. The variation in the response pattern of salivary PDGF-AB and PDGF-DD, compared to other studied salivary growth factors, may be attributed to their unique roles and regulatory mechanisms in relation to diabetes. Unlike PDGF-AB and PDGF-DD, PDGF-CC is heavily implicated in angiogenesis, processes that are often dysregulated in diabetes (40). The distinctive role of PDGF-CC in the diabetic environment highlights its potential as both a specific biomarker among PDGFs and a therapeutic target.

A previous study found that a reduction in PDGF-BB expression has been observed in diabetic patients, which negatively impacts angiogenesis and wound healing (41). Additionally, Tyndall et al. reported significantly reduced PDGF levels in diabetic fracture callus at 2- and 4-days post-fracture (42). Conversely, a study has demonstrated that plasma levels of PDGF-BB are elevated in cases of diabetic foot syndrome and correlate with the severity of the condition, suggesting a role for PDGF-BB in the body's response to the ischemic and hypoxic conditions associated with diabetes (43). Despite these findings, the relationship between PDGF levels and diabetic conditions remains complex and somewhat inconsistent. For instance, one study reported no significant differences in serum PDGF levels between diabetic patients and controls, with 23% of diabetic patients exhibiting levels above the control range (44). Another study found elevated serum PDGF levels in patients with uncomplicated diabetes compared to controls, suggesting a potential link between PDGF and diabetic pathology (45). Contrary, PDGF in vitreous or serum did not correlate significantly with HbA1c levels, age, or gender (46, 47). This inconsistency in PDGF levels may be explained by differences in the PDGF subtypes examined, the site of expression, and the methodologies employed. Similarly, in our current research, we observed varying responses and salivary levels across the three studied PDGF subtypes.

Local delivery of PDGF has been shown to enhance wound healing in patients with poorly controlled diabetes, highlighting its potential role in improving healing processes under diabetic conditions (48). Additionally, studies have indicated that PDGF and FGF can reverse impaired healing in protein-malnourished diabetic mice (49).

### Clinical significance, strengths, and limitations

The current study focuses on the salivary levels and response of growth factors during wound healing in extraction sockets of diabetic patients. Optimizing the growth factor response in impaired wound healing and comparing it to that in healthy individuals could pave the way for utilizing these growth factors as therapeutic agents, potentially for topical application in orally compromised wounds. The use of topical growth factors to enhance wound healing in diabetic patients is a promising therapeutic strategy, given the complex nature of diabetic wounds and their resistance to conventional treatments. Growth factors play a crucial role in the wound healing process by modulating cellular activities such as proliferation, migration, and angiogenesis. Various growth factors have been studied for their potential to improve healing outcomes in diabetic wounds. Interestingly, innovative delivery systems, such as EGF-loaded chitosan nanoparticles in thermos-responsive hydrogels, have shown promise in enhancing wound healing by reducing inflammation and promoting collagen synthesis in diabetic models (50). Additionally, recombinant EGF and recombinant PDGF significantly enhanced the healing rate of diabetic foot ulcers when used as adjuncts to standard care, with recombinant EGF potentially outperforming other growth factors (51). Nevertheless, PDGF enhances the cellular activities necessary for wound repair, such as fibroblast proliferation and collagen deposition (52). Additionally, topical insulin has been explored as a cost-effective option, showing potential for modulating inflammation and enhancing healing (53).

Despite the promising results, the application of growth factors in diabetic wound healing faces challenges such as cost, stability, and variability in patient response. Future research should focus on optimizing delivery systems and exploring combinations of therapies to enhance the efficacy and accessibility of these treatments. Additionally, understanding the molecular mechanisms underlying growth factor action can lead to more targeted and effective interventions for diabetic wound care.

A key strength of this study is that it is the first to identify and compare salivary growth factors in diabetic individuals vs. healthy controls, as well as to assess the response of these growth factors following oral wounds. However, the control group was smaller than the diabetes group, which may have reduced the precision of between-group estimates. Accordingly, these findings should be interpreted in the context of the sample size and confirmed in larger cohorts. In addition, the control and type 2 diabetes groups differed in mean age, with the diabetic cohort being older than the control group. This age difference is a study limitation that may have introduced some variability between groups and could affect comparisons. The short follow-up period of two days post-extraction may not reflect the full healing process or temporal biomarker changes. Further patient-related data, such as BMI, should also be considered in future studies. Expanding the follow-up period and incorporating immunological and omics analyses across groups with different HbA1c levels are recommended to better elucidate the molecular mechanisms and optimize growth factor applications for enhancing socket healing in diabetic patients. Finally, because our cohort included only well-controlled type 2 diabetes patients, the findings may not generalize to poorly controlled diabetes or to individuals with microvascular complications, in whom wound healing impairment is typically more pronounced.

## Conclusion

Salivary growth factor levels are generally lower in individuals with type 2 diabetes and show a different response to tooth extraction compared to healthy individuals. This discrepancy may account for the unique oral wound healing process observed in diabetic patients. Larger studies with balanced group recruitment and longer follow-up, incorporating standardized clinical wound-healing assessments, are needed to clarify the clinical relevance of the observed biomarker changes in oral wounds among patients with diabetes.

## Data Availability

The datasets presented in this study can be found in online repositories. The names of the repository/repositories and accession number(s) can be found below: DOI:10.17632/bs5b9gyv4c.1.
